# Correlation between immune biomarkers and surrogate markers of severity of Acute Pancreatitis in an HIV endemic region

**DOI:** 10.1016/j.pan.2025.11.014

**Published:** 2025-11-21

**Authors:** F. Madela, K. Chiliza, X. Mfeka, K. Gopee, M. Marin, M. Kongstad, S. Ostrowski, M. Ghanizada, H. Kløverpris, S. Thomson, C. Aldous

**Affiliations:** a University of KwaZulu Natal, Department of Surgery, Durban, South Africa; b Department of Education, KwaZulu-Natal, South Africa; c African Health Research Institute (AHRI), Durban, South Africa; d Department of Clinical Immunology, Copenhagen University Hospital, Rigshospitalet, Copenhagen, Denmark; e Department of Immunology and Microbiology, University of Copenhagen, Copenhagen, Denmark; f School of Laboratory Medicine and Medical Sciences, University of KwaZulu-Natal, Durban, South Africa; g Division of Gastroenterology, Department of Medicine, University of Cape Town, South Africa; h School of Clinical Medicine, University of KwaZulu-Natal, Durban, South Africa

**Keywords:** Acute pancreatitis, Cytokines, HIV, Severity, Biomarker, Revised Atlanta classification

## Abstract

**Background:**

Acute pancreatitis (AP) varies in severity, and traditional severity stratifications often fail to predict its early course. In people living with HIV (PLWH), chronic immune dysregulation leads to aberrant cytokine responses which influence biomarker performance. We hypothesize that in regions with high prevalence of HIV infection, HIV status will influence the immune biomarker performance in the prediction of severity of AP.

**Methodology:**

In this prospective case-control study conducted in KwaZulu-Natal, 144 adult patients with AP (29 % PLWH) were enrolled. Blood samples were collected within 24 h of admission, and plasma was isolated and cryopreserved before cytokine levels were quantified using a multiplex electro-chemiluminescence assay. Clinical severity was assessed using revised Atlanta classification. Receiver operating characteristic analyses were performed to derive optimal biomarker thresholds based on Youden’s index, with significance set at p < 0.01.

**Results:**

In people without HIV, IL-6, TNF-α, IL-15, IL-17, and MCP-1 were significantly upregulated in severe AP, with IL-15 demonstrating the highest discriminative performance (AUC 0.917; optimal cut-off 3.79 pg/mL; sensitivity 94.1 %, specificity 72.9 %). In PLWH patients, TNF-α was the only cytokine significantly associated with AP severity (AUC 0.974; optimal cut-off 9.42 pg/mL; sensitivity 100 %, specificity 97.4 %), while IL-17 did not predict severity in PLWH. Across the combined cohort, cytokine thresholds were higher in PLWH.

**Conclusion:**

Distinct immune signatures correlate with AP severity and are significantly influenced by HIV status. IL-15 outperformed IL-6, TNF-α, IL-17, and MCP-1 in people without HIV, whereas TNF-α was the only predictor of severity in PLWH. Higher thresholds for prediction of severity in PLWH underscore the need for HIV-specific biomarker thresholds and further research into tailored severity stratification, and development of immunomodulating therapies in AP.

## 1 Introduction

Acute pancreatitis (AP) is a common gastrointestinal condition with heterogenous severity [1]. Mild AP is typically self-limiting whereas severe AP has a high morbidity and high mortality of up to 30 % [2,3]. Early severity stratification during admission is crucial for influencing prognosis and patient outcomes [4]. The Revised Atlanta Classification (RAC) is the most recognised system for severity stratification of AP [5]. The system stratifies severity of AP into mild, moderately severe, and severe AP based on the modified Marshall scoring system for organ dysfunction [6]. While most AP cases are mild, 23 % are moderately severe and 12–20 % are severe [2,7]. Persistent organ dysfunction is a key determinant of adverse outcomes compared to the extent of local complications of the pancreas [8]. RAC is considered currently the best system in identifying severe AP, and has superseded earlier severity stratification systems which have extremely variable sensitivities and specificities and are often temporally dependant. More recently, focus has turned to the molecular basis of how the systemic inflammatory response is mediated as a method to improve severity stratification [8–10].

Three pathways are believed to be responsible for the specific immune response observed in the systemic injury associated with severe AP [11]. The first is the DAMPs-NRLP3-inflammasome pathway leading to both systemic and local tissue damage [11]. The second pathway is the capillary leak syndrome resulting from excessive vascular permeability due to systemic inflammation [11]. The last pathway involves mitochondrial dysfunction that follows release of toxic unsaturated free fatty acids into the systemic circulation [11]. The systemic inflammation leading to organ dysfunction in AP results from cascade activation of cytokines and chemokines due to local pancreatic inflammation [12].

A recent study found seven cytokines (IL-1β, IL-5, IL-6, IL-8, IL-10, IL-17, and TNF-α) to be linked to severe AP within the first 24 h of admission [3]. Upregulation of anti-inflammatory cytokine IL-10 is associated with a low risk of developing severe AP [13]. Recent research has shown that a multi-cytokine panel offers a superior sensitivity and specificity for early prediction of severe AP compared to individual cytokines or traditional surrogate stratification systems [14].

The hallmark of untreated HIV is dysregulated cytokine production with elevated pro-inflammatory cytokines (TNF-α, IL-6, and IL-1β), and upregulation of anti-inflammatory cytokines (IL-10) [15]. The phenotype of impaired cytokine production due to innate immune system dysregulation in people living with HIV (PLWH) is altered function of monocytes, natural killer (NK) cells, and dendritic cells (DCs), leading to inefficient T cells stimulation, and fewer gamma-delta T (γδ T) cells [16]. Despite antiretroviral therapy (ART), sub-optimal immune reconstitution and chronic inflammation persist, with up to 30 % of PLWH showing modest CD4^+^ T-cell improvements [15,17].

It is postulated that PLWH might exhibit an attenuated immune response to AP, given their cytokine dysregulation. Investigation of how immune dysfunctions in PLWH affect AP outcome, could guide tailored clinical management strategies in HIV-endemic regions. Previous studies on cytokine correlation with severity of AP excluded PLWH [3]. The current study aimed to prospectively assess the correlation between immune biomarkers with severity of AP in an HIV endemic region.

## 2 Methods

The study was conducted over a six-month period from December 2023 to May 2024 at five public sector hospitals (Addington, Dr Pixley KaIsaka Seme Memorial, Grey’s, Prince Mshiyeni Memorial, and Victoria Mxenge Memorial) in KwaZulu-Natal (KZN), which is a largely rural province with the second largest population of 11.5 million and the highest prevalence of HIV in South Africa.

### 2.1 Study design and population

This was a cross-sectional, prospective, case-control study to assess cytokine response by HIV status in patients presenting with AP. Written informed consent was obtained from participants and whole blood collected within 24 h of admission with AP for cytokine levels, HIV test, viral load, and CD4^+^ count. We included patients who presented within five days from onset of symptoms. Patients who refused to be tested for HIV or known with chronic pancreatitis were excluded from the study. Demographic, and clinical data including HIV status, CD4^+^ count, viral loads, severity stratification of AP using Revised Atlanta classification (2012 edition) were pseudonymously recorded in an online database, Research Electronic Data Capture (RedCap^R^). The diagnosis of AP was by two out of three parameters of standard clinical criteria, namely typical abdominal pain, a serum amylase or lipase level of ≥3 times the upper limit of normal, or evidence of AP on imaging.

### 2.2 Plasma and peripheral blood mononuclear cell (PBMC) isolation

Whole blood samples were drawn intravenously into 10 ml ethylenediaminetetraacetic acid (EDTA) tubes, transported and processed within 2 h of collection. PBMCs were isolated from whole blood by Histopaque 1077 (Sigma-Aldrich) density gradient centrifugation at 1500*g* for 15 min at room temperature in Leu-cosep tubes (Greiner Bio-One, 163290P). Following centrifugation, the plasma was collected, aliquoted, and stored at −80 °C until cytokine analysis.

### 2.3 Quantification of cytokine levels in plasma

Plasma cytokine concentrations were measured using the Mesoscale Discovery (MSD) platform with the V-PLEX Human Cytokine 30-Plex Panel (K15054G – Eotaxin-1, Eotaxin-3, GM-CSF, IFN-γ, IL-1α, IL-1β, IL-2, IL-4, IL-5, IL-6, IL-7, IL-8, IL-8 (HA), IL-10, IL-12p70, IL-12/IL-23p40, IL-13, IL-15, IL-16, IL-17A, IP-10, MCP-1, MCP-4, MDC, MIP-1α, MIP-1β, TARC, TNF-α, TNF-β, VEGF-A) and R-PLEX Human Antibody-set (CD14, E-cadherin, FABP2, LBP, RAGE, Reg-3-alpha, PAF-acetyl-hydrolase) (Mesoscale Discovery, Rock-ville, MD). The MSD assay was conducted with investigators and technicians blinded to the participant data. The laboratory received 700 μL of each plasma sample. In preparation for the assay, the lyophilized cytokine standards and controls were reconstituted with the provided assay diluents. The MSD assay was performed according to the manufacturer’s instructions. The electrochemiluminescence (ECL) signal was detected and imaged by the MESO QuickPlex SQ 120 plate reader. The concentration of each sample was determined using a four-parameter logistic curve fitting model, which was generated from the standards. The concentrations were then calibrated according to the certificate of analysis provided by MSD.

### 2.4 Statistical analysis

IBM SPSS version 29 was used to analyze the data. Measured continuous variables were checked for normality of distribution using the Shapiro-Wilks test. Non normally distributed variables were summarized and presented using median and inter-quartile range. Normally distributed variables were presented using mean and standard deviation. Non normal distributions were compared between three groups using Kruskal-Wallis tests and between two groups using Mann-Whitney U tests. The acquired cytokine concentrations (pg/ml) were analysed using the R statistical programming language. The Wilcoxon-test paired *t*-test was used to compare two paired groups. Correlations between non normally distributed variables were assessed using Spearman’s correlation coefficients. Receiver operating characteristic (ROC) curves were used to find optimal cut points for the cytokine variables which were statistically significantly associated with severe disease by HIV status. The cut point with the highest Youden’s index was chosen as the optimal cut point. ROC curves (area under the curve) AUC were compared using the roccomp command in Stata version 18 by Chi squared tests. Individual ROC curves were done for each biomarker, but graphed in a combined ROC curve. Due to the number of statistical tests performed, p values < 0.01 were considered as statistically significant. For overall comparisons which were statistically significant at the 0.01 level, Bonferroni adjusted post hoc tests were done for comparison.

#### Ethical approval

Ethical approval for the analysis was obtained from the Biomedical Research Ethics Committee of the University of Kwazulu-Natal, BREC/407/2019. Permission to conduct the study at the respective hospitals was granted by the Research Committee of the KZN Provincial Department of Health (NHRD Ref: KZ_201911_015)

## 3 Results

### 3.1 Patient characteristics

156 participants and 28 controls were enrolled to the study. Twelve were excluded due to declining consent for an HIV test (3), failure to meet diagnostic criteria (3), and for being included in the pilot stage of the study and analysed using a different protocol (6). The remaining 144 participants were included for analysis. Table 1 shows the characteristics, aetiology, and severity stratification of the cohort by HIV status. Severe AP accounted for 38 (14.6 %) of cases. Alcohol-induced AP (51.4 %) and biliary AP (34 %) were the major aetiologies. People living with HIV numbered 42 (29.17 %) of whom 35 were on ART and 34 were virally suppressed. Three patients on ART had severe disease, while only one ART naïve among seven viraemic PLWH had severe disease. The male to female ratio was 57:45 in people without HIV and 19:23 in the cohort of PLWH with similar age (mean of 42 years) in both groups.

Table 2 is a redacted table of comparisons of cytokines between RAC categories by HIV status (For a complete table of the entire panel of biomarkers, see supplementary tables). For people without HIV, there was a statistically significant difference at the 0.01 level in cytokine levels between the three groups for IL-6, IL-15, IL-17, TNF-α and MCP-1. For PLWH, there was a statistically significant difference at the 0.01 level in cytokine level between the three groups for TNF-α. Overall, the majority of significant differences was found between mild and severe groups when pairwise adjusted tests were done.

**Fig. 1:**
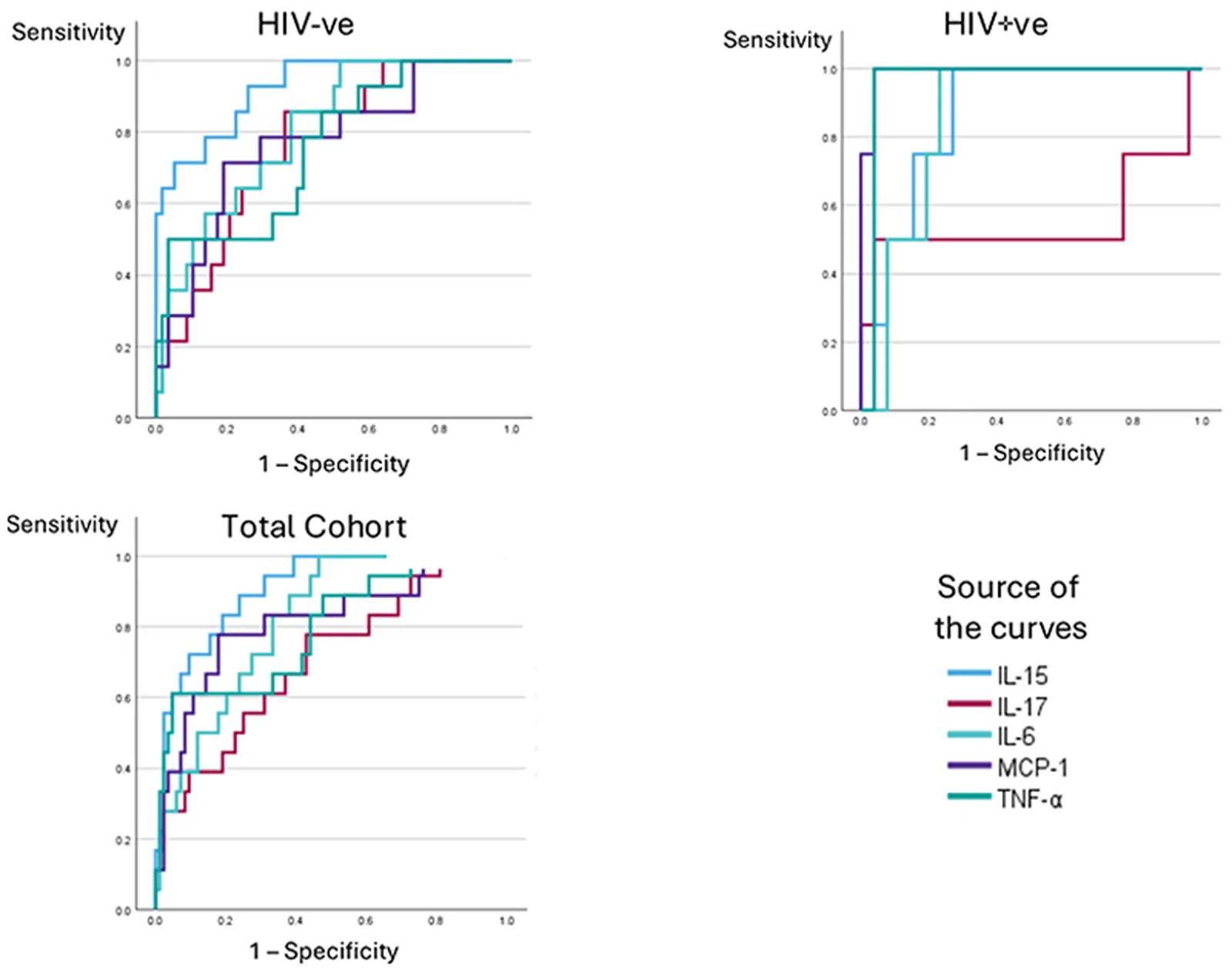
Biomarker ROC curves for severe disease.

Fig. 1 shows the ROC curves of significant biomarkers for cut offs to predict severe disease according to Revised Atlanta Classification. Table 3 is a summary of cut points of biomarkers based on the highest Youden’s index.

Comparisons between the 5 ROC curves in HIV negative group: The null hypothesis is that the AUC of all 5 curves is equal. Chi square (4 degrees of freedom) = 9.04, p = 0.0604. There was no difference between all 5 curves.

Pairwise comparison between IL 15 (highest AUC) and each curve:

IL-15 vs IL-17: p = 0.0254.
IL-15 vs IL-6: p = 0.1022.
IL-15 vs TNF-α: p = 0.0085.
IL-15 vs MCP-1: 0.0249.

Therefore at the 0.01 level of significance, only TNF-α was a worse predictor than IL-15.

Comparisons between the 4 ROC curves in HIV positive group: The null hypothesis is that the AUC of all 4 curves is equal. Chi square (3 degrees of freedom) = 5.66, p = 0.1293. There was no difference between all 4 curves.

Pairwise comparison between TNF-α (highest AUC) and each curve:

IL-6 vs TNF-α p = 0.0371.
IL-15 vs TNF-α: p = 0.2129.
MCP-1 vs TNF-α: p = 0.4951.
IL-17 vs TNF-α: p = 0.1087.

Therefore at the 0.01 level of significance, all curves were as good as TNF-α.

Comparisons between the 5 ROC curves in total cohort:

The null hypothesis is that the AUC of all 5 curves is equal. Chi square (4 degrees of freedom) = 8.48, p = 0.0754. There was no difference between all 5 curves.

Pairwise comparison between IL-15 (highest AUC) and each curve:

IL-6 vs IL-15 p = 0.0730.
TNF-α vs IL-15 p = 0.0276.
MCP-1 vs IL-15 p = 0.0513.
IL-17 vs IL-15 p = 0.0120.

Therefore at the 0.01 level of significance, all curves were as good as IL-15.

## 4 Discussion

In this study of 144 participants, alcohol-induced AP emerged as the primary cause (51.4 %) of the majority of cases presenting with AP. There were 42 (29.17 %) PLWH with the majority of patients on ART (83 %), and virally suppressed (81 %). Most cases were mild, with only 14.6 % (16.7 % of people without HIV and 9.5 % PLWH) being severe based on RAC as a surrogate stratification of severity. In severe AP, four cytokines and one chemokine from a panel of 27 biomarkers (IL-6, TNF-α, IL-15, IL-17, MCP-1) were significantly upregulated than in mild cases in people without HIV, whereas only TNF-α was significantly upregulated than in mild cases in PLWH. These five immune biomarkers were significantly correlated with severity in people without HIV, but IL-17 did not predict severity in PLWH. IL-15 showed the highest AUC with high sensitivity and specificity in people without HIV and combined cohorts, while TNF-α showed the highest AUC with high sensitivity and specificity in PLWH. This study also revealed that optimal cut off point of immune biomarkers for correlation with severity were higher in PLWH based on the highest Youden’s index than in people without HIV despite a lower than expected proportion of severe cases (9.5 %).

While alcohol-induced AP constituted the majority of cases in our cohort, it was not a predictive factor for severity of disease. Research has not demonstrated a correlation between cytokine dysregulation in severe disease with the aetiology of AP [18]. This is because, regardless of diverse triggers for AP, there are common pathways that underscore aetiopathogenesis of severe AP secondary to ductal and acinar cell injury [11,19].

Several studies have identified TNF-α, IL-6, MCP-1 to be correlated with organ dysfunction and severity in AP [3,9,13,18,20]. Additionally, a few have linked IL-15, and IL-17 with severe AP [3,21,22]. However, none of the studies included PLWH. TNF-α peaks early in the SIRS, and triggers the release of IL-1β, IL-6, and IL-8 resulting in a cytokine storm [23]. IL-6 is a pleiotropic pro-inflammatory cytokine involved in the production of C-reactive protein (CRP) in the liver, and the differentiation of IL-17 producing T-helper cells [11]. Monocyte chemoattractant protein-1 (MCP-1) is a chemokine involved in leucocyte migration and vascular permeability [11]. Interleukin-15 is a cytokine involved in the Th1 immune response. It is structurally similar to IL-2 receptor but secreted by monocytes/macrophages instead of T cells, and leads to T helper cell proliferation [24]. Interleukin-17, a pro-inflammatory cytokine released by T helper 17 (Th17), γδ T, NK, and dendritic cells of the innate immune system, augments the inflammatory cascade of AP induced by other cytokines, such as IL-6, IL-8, and TNF-α [21]. Early upregulation of IL-17 is linked with significant morbidity and mortality in severe AP [25].

Studies have found a wide variability in the cut-off levels of immune biomarkers, particularly IL-6, for prediction AP severity [3,9,13,14,26]. Several factors contribute to this inconsistency, including the retrospective and exploratory nature of some studies, short duration of production and rapid liver clearance of cytokines, and selection bias due to the proportion of severe AP cases included to establish cut-off values [27,28]. Some studies include a higher percentage of severe AP cases than the 12–20 % typically reported in the literature. Since cut-off values are derived from the median biomarker levels, cohorts with a higher representation of severe cases tend to have high median values, and project higher cut-off thresholds [28]. A recent study evaluating the predefined cut-off levels of common biomarkers for predicting severe AP based on previous research found that these thresholds were challenging to apply to populations different from the original study cohorts [28]. The TNF-α, IL-15, and IL-17 threshold ranges in our study were not dissimilar to previous observations, but the cut-offs for IL-6 and MCP-1 reflect the same lack of consistency or consensus on the optimal cut points. Due to the significant inter-observer variability in immune biomarker performance, immune signatures that reflect multiple points in the pathways of severe AP are likely to outperform individual cytokines levels or traditional surrogate severity stratifications in the severity predictive accuracy [14].

In our study, inter-related immune biomarkers (TNF-α with IL-6/IL-17, and MCP-1 with IL-15) were upregulated in severe AP, and IL-17, released by impaired or altered cells (Th17, γδ T, NK, and DCs) in the HIV innate immune system phenotype, was not predictive of severity of AP in PLWH [16]. In PLWH, systemic inflammatory response syndrome (SIRS) has been observed to be attenuated, and to be an ineffective predictor of severity of AP [29]. In a retrospective study, immunocompromised PLWH, based on CD4^+^ lymphocytes of less than 200, were found to have worse mortality outcomes than immunocompetent patients secondary to infectious causes [30]. Due to the retrospective nature of that study, it was not possible to determine if the reported mortalities were early or late on the bimodal pattern of mortality in AP. However, the authors concluded that mortality was related to infections as opposed to cytokine storm which is a feature of early mortality. Our study found higher immune biomarker thresholds linked with severity in AP among chronic HIV-infected patients, even though the majority (more than 80 %) were virally suppressed. This could represent failure of immune reconstitution possibly due to delayed initiation of ART. It also suggests that HIV, irrespective of ART, may blunt the effects of cytokine storm in the early phase of severe AP.

Future management of severe AP should prioritize the early identification of upregulated immune biomarkers that are associated with severe AP in both PLWH and people without HIV. Precision immunomodulation therapy should be customized to target these biomarkers. The objective of this approach is to arrest the insults of DAMPs-NLRP3 inflammasome and capillary leak syndrome pathways, which are key factors in severe AP and the early mortality within the bimodal pattern of mortality in AP.

### 4.1 Strengths and limitations of the study

This is a cross-sectional, prospective case-control study. A few previous studies that investigated correlation of immune bio-markers with severity of AP were either retrospective or did not consider HIV status. Unlike many similar studies where patients presented within 48 h of onset of symptoms, several of our participants experienced delays in diagnosis, admission or referral. This may lead to heterogenous values of cytokines due to their temporal profile. Another limitation is that too few of our PLWH had severe disease.

## 5 Conclusion

We identified a cytokine panel of IL-6, TNF-α, IL-15, IL-17, and MCP-1 to be correlated with severity of AP from a panel of 27 biomarkers. TNF-α had the highest AUC in PLWH, and IL-15 in people without HIV. IL-17, while it is a severity prognostic factor in people without HIV, it was not found to be predictive of severity in PLWH. Cut-off points correlating with severity of AP had higher thresholds in PLWH than in people without HIV, suggesting the need for further research to tailor the management of severe disease in PLWH with respect to immune signatures and thresholds that predict severity in this cohort of patients.

## Supplementary Material

Supplementary data to this article can be found online at https://doi.org/10.1016/j.pan.2025.11.014.

Multimedia component 1

Multimedia component 2

## Figures and Tables

**Table 1:** Characteristics of patients with Acute Pancreatitis by HIV status.

	HIV status					
	Negative (n = 102)		Positive (n = 42)		Total n = 144)	
	Mean	SD	Mean	SD	Mean	SD
Age	42	15	42	10	42	14
	n	%	n	%	n	%
Sex						
Male	57	55.9	19	45.2	76	52.8
Female	45	44.1	23	54.8	68	47.2
Aetiology						
Alcohol	51	50.0	23	54.8	74	51.4
Gallstones	35	34.3	14	33.3	49	34.0
ERCP	5	4.9	0	0.0	5	3.5
HIV related	0	0.0	4	9.5	4	2.8
Idiopathic	4	3.9	0	0.0	4	2.8
Malignancy	1	1.0	0	0.0	1	0.7
HTG	4	3.9	1	2.4	5	3.5
Drugs	2	2.0	0	0.0	2	1.4
RAC																																																																																						
Mild	78	76.5	33	78.6	111	77.1
Moderate	7	6.9	5	11.9	12	8.3
Severe	17	16.7	4	9.5	21	14.6

HTG: Hypertriglyceridaemia RAC: Revised Atlanta Classification.

**Table 2:** Comparison of cytokines between Revised Atlanta categories by HIV status.

		HIV Negative				HIV Positive			
		Mild	Mod	Severe	p-value	Mild	Mod	Severe	p-value
IL-10	Valid N	78	7	17	0.054	33	5	4	0.013
	Median	0.77	1.12	1.75		0.52	0.71	4.05	
IL-6	Valid N	78	7	17	<0.001	33	5	4	0.141
	Median	6.88	10.55	67.21	Mild vs severe p < 0.001	5.45	9.91	22.73	
IL-8_LA	Valid N	78	7	17	0.013	33	5	4	0.031
	Median	10.43	9.60	20.45		8.33	14.76	29.85	
TNF-α	Valid N	78	7	17	0.030	33	5	4	0.008
	Median	2.11	2.10	3.02	Mild vs severe p = 0.008	2.36	1.69	12.91	Mild vs severe p = 0.003 Mod vs severe p = 0.007
IL-15	Valid N	78	7	17	<0.001	33	5	4	0.012
	Median	2.73	6.50	8.74	Mild vs severe p < 0.001	3.24	4.11	7.71	
MCP-1	Valid N	78	7	17	0.002	33	5	4	0.015
	Median	83.27	96.32	166.23	Mild vs severe p < 0.001	90.06	80.91	228.22	
IL-17	Valid N	53	5	14	<0.001	23	3	4	0.934
	Median	1.75	6.82	4.44	Mild vs severe p < 0.001 Mild vs mod p = 0.006	3.79	2.22	8.17	

* Valid N refers to the number of specimens whose samples were analysable. **mod is moderate.

**Table 3:** Biomarker cut points by HIV status derived from ROC curves (*pg/mL).

Biomarker By HIV status	AUC (95 % CI)	p-value	*Cut Point	Youden’s Index	Sensitivity	Specificity
IL-6 Neg	0.819 (0.720–0.918)	<0.001	7.52	0.518	100 %	51.8 %
IL-6 Pos	0.789 (0.628–0.951)	0.059	10.19	0.658	100 %	65.8 %
IL-6 total	0.817 (0.733–0.900)	<0.001	7.52	0.537	100 %	53.7 %
TNF-α Neg	0.700 (0.554–0.845)	0.001	2.765	0.306	64.7 %	65.9 %
TNF-α Pos	0.974 (0.922–1.000)	0.002	9.42	0.974	100 %	97.4 %
TNF-α total	0.746 (0.616–0.876)	<0.001	2.54	0.380	76.2 %	61.8 %
IL-15 Neg	0.917 (0.853–0.981)	<0.001	3.79	0.671	94.1 %	72.9 %
IL-15 Pos	0.888 (0.766–1.010)	0.011	4.56	0.763	100 %	76.3 %
IL-15 total	0.907 (0.851–0.964)	<0.001	4.33	0.677	90.5 %	77.2 %
MCP-1 Neg	0.768 (0.640–0.897)	0.001	127.4	0.541	76.5 %	77.6 %
MCP-1 Pos	0.941(0.864–1.000)	0.004	173.9	0.868	100 %	86.8 %
MCP-1 total	0.794 (0.682–0.905)	0.001	127.4	0.566	81 %	75.6 %
IL-17 Neg	0.767 (0.641–0.893)	0.002	3.176	0.421	71.4 %	70.7 %
IL-17 Pos	0.558 (0.069–1.000)	0.0714	N/A	N/A	N/A	N/A
IL-17 total	0.707 (0.571–0.843)	0.006	2.65	0.349	77.8 %	57.1 %
