# Supplementary material for: Correlation between immune biomarkers and surrogate markers of severity of Acute Pancreatitis in an HIV endemic region
Source: Pancreatology. Author manuscript; Available in PMC 2026 Oct 15. (PMC7619553; doi:10.1016/j.pan.2025.11.014)
Supplement: Supplementary file 1 [file EMS219698-supplement-Multimedia_component_1.docx]

**Suppl Table 2: Comparison of cytokines between Revised Atlanta categories by HIV status**

|  | | HIV status | | | | | | | |
| --- | --- | --- | --- | --- | --- | --- | --- | --- | --- |
|  |  | Negative | | | | Positive | | | |
|  |  | Revised Atlanta classification | | | | Revised Atlanta classification | | | |
|  |  | Mild | Moderate | Severe | p-value | Mild | Moderate | Severe | p-value |
| FABP2 | Valid N | 78 | 7 | 17 | 0.172 | 33 | 5 | 4 | 0.088 |
|  | Median | 331.53 | 327.27 | 198.37 |  | 497.31 | 154.19 | 466.37 |  |
| CD14 | Valid N | 73 | 7 | 17 | 0.011 | 30 | 5 | 4 | 0.481 |
|  | Median | 1135017.74 | 1271489.70 | 1796686.93 |  | 1818548.49 | 1821873.35 | 2550481.85 |  |
| IFN-γ | Valid N | 76 | 7 | 15 | 0.878 | 32 | 5 | 4 | 0.879 |
|  | Median | 3.30 | 3.80 | 2.15 |  | 6.43 | 8.00 | 12.23 |  |
| IL-10 | Valid N | 78 | 7 | 17 | 0.054 | 33 | 5 | 4 | 0.013 |
|  | Median | 0.77 | 1.12 | 1.75 |  | 0.52 | 0.71 | 4.05 |  |
| IL-12p70 | Valid N | 74 | 6 | 12 | 0.708 | 24 | 5 | 2 | 0.539 |
|  | Median | 0.34 | 0.19 | 0.32 |  | 0.26 | 0.12 | 0.18 |  |
| IL-1β | Valid N | 76 | 7 | 17 | 0.477 | 32 | 5 | 4 | 0.620 |
|  | Median | 0.42 | 0.45 | 0.70 |  | 0.36 | 0.77 | 0.45 |  |
| IL-2 | Valid N | 77 | 6 | 16 | 0.840 | 33 | 5 | 4 | 0.570 |
|  | Median | 0.47 | 0.58 | 0.62 |  | 0.41 | 0.79 | 0.67 |  |
| IL-6 | Valid N | 78 | 7 | 17 | <0.001 | 33 | 5 | 4 | 0.141 |
|  | Median | 6.88 | 10.55 | 67.21 | Mild vs severe p<0.001 | 5.45 | 9.91 | 22.73 |  |
| IL-8_LA | Valid N | 78 | 7 | 17 | 0.013 | 33 | 5 | 4 | 0.031 |
|  | Median | 10.43 | 9.60 | 20.45 |  | 8.33 | 14.76 | 29.85 |  |
| TNF-α | Valid N | 78 | 7 | 17 | 0.030 | 33 | 5 | 4 | 0.008 |
|  | Median | 2.11 | 2.10 | 3.02 | Mild vs severe p=0.008 | 2.36 | 1.69 | 12.91 | Mild vs severe p=0.003 |
|  |  |  |  |  |  |  |  |  | Moderate vs severe p=0.007 |
| IL-12 | Valid N | 78 | 7 | 17 | 0.790 | 33 | 5 | 4 | 0.814 |
|  | Median | 55.15 | 46.11 | 50.59 |  | 120.49 | 93.50 | 117.98 |  |
| IL-15 | Valid N | 78 | 7 | 17 | <0.001 | 33 | 5 | 4 | 0.012 |
|  | Median | 2.73 | 6.50 | 8.74 | Mild vs severe p<0.001 | 3.24 | 4.11 | 7.71 |  |
| IL-16 | Valid N | 78 | 7 | 17 | 0.033 | 33 | 5 | 4 | 0.366 |
|  | Median | 159.21 | 193.52 | 487.85 |  | 236.10 | 721.02 | 369.40 |  |
| IL-5 | Valid N | 77 | 6 | 17 | 0.768 | 33 | 5 | 4 | 0.848 |
|  | Median | 0.53 | 0.63 | 0.48 |  | 0.62 | 1.29 | 1.34 |  |
| IL-7 | Valid N | 78 | 7 | 17 | 0.633 | 33 | 5 | 4 | 0.612 |
|  | Median | 3.17 | 3.44 | 1.71 |  | 6.72 | 10.35 | 6.42 |  |
| TNF-β | Valid N | 72 | 6 | 14 | 0.077 | 31 | 3 | 2 | 0.362 |
|  | Median | 0.19 | 0.22 | 0.10 |  | 0.21 | 0.13 | 0.30 |  |
| VEGF_cyto | Valid N | 78 | 7 | 17 | 0.192 | 33 | 5 | 4 | 0.756 |
|  | Median | 36.76 | 88.73 | 60.99 |  | 78.26 | 83.33 | 33.70 |  |
| Eotaxin-1 | Valid N | 77 | 7 | 16 | 0.043 | 33 | 5 | 4 | 0.266 |
|  | Median | 159.36 | 241.54 | 202.63 |  | 227.20 | 257.22 | 392.85 |  |
| Eotaxin-3 | Valid N | 78 | 7 | 17 | 0.314 | 33 | 5 | 4 | 0.943 |
|  | Median | 13.60 | 16.48 | 19.73 |  | 12.25 | 18.14 | 9.91 |  |
| IP-10 | Valid N | 78 | 7 | 17 | 0.030 | 33 | 5 | 4 | 0.184 |
|  | Median | 252.21 | 263.72 | 529.51 |  | 523.74 | 635.26 | 1261.71 |  |
| MCP-1 | Valid N | 78 | 7 | 17 | 0.002 | 33 | 5 | 4 | 0.015 |
|  | Median | 83.27 | 96.32 | 166.23 | Mild vs severe p<0.001 | 90.06 | 80.91 | 228.22 |  |
| MCP-4 | Valid N | 78 | 7 | 17 | 0.450 | 33 | 5 | 4 | 0.954 |
|  | Median | 44.75 | 48.82 | 36.82 |  | 79.54 | 88.55 | 78.19 |  |
| MDC | Valid N | 78 | 7 | 17 | 0.261 | 33 | 5 | 4 | 0.407 |
|  | Median | 623.68 | 743.78 | 469.33 |  | 780.62 | 811.15 | 673.01 |  |
| MIP-1α | Valid N | 74 | 7 | 16 | 0.324 | 31 | 5 | 4 | 0.098 |
|  | Median | 10.96 | 6.89 | 12.84 |  | 14.61 | 11.43 | 24.47 |  |
| MIP-1β | Valid N | 78 | 7 | 17 | 0.376 | 33 | 5 | 4 | 0.230 |
|  | Median | 58.42 | 40.12 | 60.40 |  | 75.73 | 81.50 | 223.95 |  |
| TARC | Valid N | 78 | 7 | 17 | 0.183 | 33 | 5 | 4 | 0.430 |
|  | Median | 69.88 | 78.79 | 41.94 |  | 123.24 | 166.47 | 107.88 |  |
| IL-17 | Valid N | 53 | 5 | 14 | <0.001 | 23 | 3 | 4 | 0.934 |
|  | Median | 1.75 | 6.82 | 4.44 | Mild vs severe p<0.001 | 3.79 | 2.22 | 8.17 |  |
|  |  |  |  |  | Mild vs moderate p=0.006 |  |  |  |  |
